# Performance d’Hemocue Hb 201+ dans le diagnostic de l’anémie de l’enfant dans les structures sanitaires du niveau périphérique au Togo

**DOI:** 10.4102/ajlm.v2i1.28

**Published:** 2013-12-18

**Authors:** Ahoefa Vovor, Ameyo Dorkenoo, Yao Layibo

**Affiliations:** 1 Faculté Mixte de Médecine et de Pharmacie, Université de Lomé, Togo; 2 Institut National d’Hygiène Lomé, Togo

## Abstract

**Contexte:**

L’anémie est un problème de santé publique dans le monde entier, et notamment dans les pays en développement. Elle a des répercussions majeures sur la santé et sur le développement économique et social d’un pays. La prise en charge des patients anémiés étant nécessaire, il faut un diagnostic biologique précis, et donc un dosage du taux d’hémoglobine par des méthodes fiables.

**Objectif:**

Évaluer les performances diagnostiques du test Hemocue Hb201+^®^.

**Méthodes:**

Étude comparative de la mesure du taux d’hémoglobine à partir du photomètre Hemocue Hb 201+^®^ et d’analyseurs d’hématologie chez 213 enfants de 6 à 59 mois souffrant d’un paludisme simple; la détermination du taux d’hémoglobine par les analyseurs est retenue comme méthode de référence pour évaluer Hemocue Hb201+^®^.

**Résultats:**

72.8% des valeurs obtenues par Hemocue Hb201+^®^ étaient à ±1 g/dl de celles de la méthode de référence. Le coefficient de corrélation de Pearson était de 0.80. La prévalence de l’anémie était de 79.3% pour la méthode de référence et de 77.9% pour Hemocue Hb201+^®^. La sensibilité et la spécificité de l’analyseur Hemocue Hb201+^®^ étaient respectivement de 95.1% et de 65.3%.

**Conclusion:**

Les résultats de l’étude ont montré que le test Hemocue Hb201+^®^ présentait une bonne sensibilité, une spécificité moyenne et une exactitude moyenne dans le diagnostic de l’anémie et dans le dosage de l’hémoglobine. Son utilisation peut être recommandée dans les structures périphériques afin de faciliter le diagnostic biologique de l’anémie et sa prise en charge dans les populations vivant dans les zones difficiles d’accès.

## Abstract (en)

**Performance of HemoCue Hb201+ in the diagnosis of anaemia in children in health facilities at the peripheral level in Togo**

**Background:**

Anaemia is a global public health problem, especially in developing countries. Anaemia has major repercussions on health status, as well as on the economic and social development of a country. Effective care of anaemic patients requires a reliable and precise diagnostic test that can determine haemoglobin levels.

**Objective:**

To evaluate the diagnostic performance of the Hemocue test Hb201+^®^.

**Method:**

This study compared haemoglobin levels measured using the photometer Hemocue Hb201+^®^ with those measured by analysers of haematology. Children aged 6 to 59 months who suffered from uncomplicated malaria were eligible for inclusion. Haemoglobin levels determined by the analysers were considered the reference for evaluation of the levels measured using Hemocue Hb201+^®^.

**Results:**

72.8% of the values obtained by Hemocue Hb201^®^ were within ±1g/dl of the reference value. The Pearson correlation coefficient was 0.80. The prevalence of anaemia was 79.3% using the reference method and 77.9% using Hemocue 201+^®^. The sensitivity and the specificity of Hemocue Hb201+^®^ were 95.1% and 65.3% respectively.

**Conclusion:**

The study results showed that the Hemocue Hb201 test+^®^ provided good sensitivity, average specificity and average precision, both for the diagnosis of anaemia and for the determination of haemoglobin levels. It may be used in peripheral centres to facilitate the laboratory diagnosis of anaemia and its management in populations that live in areas with difficult accessibility.

## Introduction

L’anémie est un problème mondial de santé publique elle touche, selon les estimations de l’Organisation mondiale de la Santé (OMS), deux milliards d’individus dans le monde. Elle est nettement plus fréquente dans les pays en voie de développement chez les enfants et les femmes où la principale étiologie est la carence en fer aggravée essentiellement par les parasitoses intestinales et le paludisme; ses conséquences sont néfastes pour le développement de l’enfant.^1^

Le diagnostic de l’anémie est déterminé par des signes cliniques et confirmé par la biologie; il repose sur le dosage de l’hémoglobine qui doit être réalisé par des méthodes et équipements fiables (spectrophotomètre de biochimie ou analyseur d’hématologie).

Au Togo, le système sanitaire est organisé de façon pyramidale selon trois niveaux: un niveau central, un niveau intermédiaire (régional) et un niveau périphérique. Si les laboratoires d’analyses médicales et biologiques des niveaux central et régional de la pyramide de santé du Togo disposent de ces équipements, beaucoup de laboratoires de biologie médicale, notamment du niveau périphérique, utilisent des méthodes de dosage du taux d’hémoglobine (méthode de Tallquist^2^ et méthode de Sahli et Gowers^3^) désuètes dont le niveau de fiabilité des résultats fournis est sujet à caution.^4^

L’hémoglobinomètre portable, Hemocue 201+^®^ (Hemocue AB, Angelholm, Suède), un appareil mesurant par photométrie le taux d’hémoglobine dans le sang, a le mérite d’être accessible et d’utilisation relativement plus facile et donc plus adapté aux réalités des laboratoires du niveau périphérique.

L’objectif de cette étude était d’évaluer les performances de l’hémoglobinomètre Hemocue Hb201+^®^ pour le diagnostic de l’anémie de l’enfant afin de le rendre disponible dans les structures sanitaires périphériques en remplacement des techniques actuellement utilisées; plus spécifiquement, il s’agissait de:

- comparer les résultats obtenus par Hemocue Hb201+^®^ et les analyseurs d’hématologie, Syxmex KX-21^®^ et Mindray BC 3000^®^.
- définir la sensibilité, la spécificité et l’exactitude d’Hemocue Hb201+^®^

## Matériel et méthodes

Il s’agit d’une étude analytique comparant la mesure du taux d’hémoglobine à partir du photomètre Hemocue Hb 201+^®^ à celle effectuée par des analyseurs d’hématologie Sysmex KX 21^®^ et Mindray BC 3000^®^.

Cette étude a été réalisée du 13 août au 14 décembre 2007. Le prélèvement et la réalisation des dosages par Hemocue Hb201+^®^ ont été réalisés sur les sites de surveillance de l’efficacité des antipaludiques à Lomé (au centre médico-social d’Adakpamé et au centre médico-social Jérusalem d’Agbalépédogan) et à Sokodé (au centre médico-social Bon Secours et à la polyclinique de Sokodé). Les patients y ont été recrutés et le dosage de l’hémoglobine à l’aide de l’hémoglobinomètre Hemocue Hb 201+^®^ y a été effectué; les dosages à l’aide des analyseurs ont été réalisés dans les laboratoires du CHU-CAMPUS à Lomé (Sysmex KX 21^®^) et du CHR de Sokodé (Mindray BC 3000^®^).

## Population étudiée

Les sujets ayant participé à la présente étude étaient des enfants âgés de 6 à 59 mois admis en consultation externe dans les structures sanitaires précitées.

### L’échantillonnage

La taille minimale de l’échantillon était de 174 enfants, calculée à partir de la formule de Schwartz: *N* = ε^2^pq/i^2^, la prévalence p de l’anémie chez l’enfant étant estimée à 87 %^5^, q = 1 – *p*, ε = 1.96 et la précision *i* = 5%.

### Critères d’inclusion

Ont été inclus les enfants âgés de 6 à 59 mois admis en consultation externe dans les centres de santé précités les jours ouvrables de 7h30 à 11h00. Ces enfants doivent répondre aux critères d’inclusion et de non inclusion définis pour l’évaluation de l’efficacité thérapeutique des combinaisons à base d’artémisinine sur sites sentinelles au Togo.^6^

Les critères d’inclusion étaient:

= être âgé de 6 mois à 6 ans
= avoir de la fièvre (température axillaire supérieure ou égale à 37.5 °C ou température rectale supérieure à 38 °C) ou rapporter un épisode de fièvre dans les 72 dernières heures
= avoir une goutte épaisse et un frottis sanguin positif.

Les critères de non inclusion étaient:

= présence d’un ou de plusieurs signes de danger ou de paludisme grave défini par l’OMS
= présence d’une malnutrition sévère (périmètre brachial < 11 cm; rapport poids/taille < 70%; œdèmes bilatéraux aux membres inférieurs)
= existence d’une autre pathologie fébrile évidente
= prise d’une quelconque médication à action antipaludique au cours des deux dernières semaines.

## Méthodes d’étude

L’échantillon biologique était constitué de sang total. Deux types de prélèvements ont été effectués: du sang capillaire prélevé au niveau de la pulpe du doigt pour le dosage sur Hemocue et 2 ml de sang veineux prélevés dans un tube contenant de l’anticoagulant EDTA pour le dosage sur les analyseurs d’hématologie. Les prélèvements sur EDTA ont été acheminés dans des glacières au Centre Hospitalier Universitaire (CHU) Campus et au Centre Hospitalier Régional (CHR) de Sokodé, structures situées à moins de 3 km des sites de prélèvement, respectivement pour les prélèvements effectués à Lomé et Sokodé.

Le dosage sur Hemocue Hb 201+^®^ a été effectué dans ces quatre centres; le dosage par l’analyseur Sysmex KX-21^®^ a été effectué au CHU Campus de Lomé pour les prélèvements provenant des deux structures sanitaires de Lomé et le dosage par l’analyseur Mindray BC 3000^®^ a été réalisé au CHR de Sokodé pour les prélèvements provenant des deux structures sanitaires de Sokodé.

Les techniciens de laboratoire ont été formés afin d’assurer la standardisation des méthodes de diagnostic (la reproductibilité interindividuelle). Un contrôle de qualité inter laboratoire a été systématiquement effectué entre le laboratoire d’hématologie du CHU Campus et celui du CHR de Sokodé pour les deux automates. Aucune différence n’a été observée entre les résultats des deux analyseurs. L’anémie a été définie par un taux d’hémoglobine inférieur à 110 g/l.

### Aspects éthiques

La clairance éthique, délivrée par le Ministère de la Santé pour la conduite des tests d’efficacité et du profil de l’hémogramme au cours de l’accès palustre simple, a couvert cette étude.

## Analyse des données

L’analyse statistique des résultats a été réalisée sur les logiciels Excel 2003 et SPSS12. Les paramètres statistiques étudiés étaient:

- Le coefficient de corrélation de Pearson pour la détermination du lien entre les valeurs obtenues par Hemocue Hb 201+^®^ et l’automate.
- Les limites de dispersion des différences entre les valeurs du taux d’hémoglobine obtenues par Hemocue 201+^®^ et celles obtenues par les analyseurs, déterminées par la méthode de Bland et Altman.^7^ Les limites de variation acceptables ont été fixées de façon arbitraire à ±10 g/l autour de la moyenne des différences entre les valeurs du taux d’hémoglobine obtenues à partir des deux méthodes. La méthode de dosage sur Hemocue Hb201+^®^ sera dite exacte lorsque les limites de dispersion des différences des valeurs des deux méthodes (moyenne ±2 SD) définies par le graphique de Bland et Altman ne dépasseront pas les limites de variation acceptables préalablement fixées.

Les performances intrinsèques (sensibilité et spécificité) et extrinsèques (valeur prédictive positive et valeur prédictive négative) du test Hemocue Hb201+^®^ par rapport à l’anémie ont été déterminées.

## Résultats

### Population d’étude

Huit cent seize enfants ont été reçus en consultation durant la période de l’étude; 340 étaient éligibles et 213 ont été inclus dans l’étude.

### Caractéristiques générales des enfants inclus dans l’étude

L’âge moyen était de 35.3 ± 14.8 mois, avec des valeurs extrêmes de 7 mois à 59 mois. Le sex-ratio était de 1.39, avec une prédominance masculine. Le poids moyen était de 12.8 ± 3 kg et la température moyenne de 38.7 ± 0.9 °C (tableau 1).

**TABLEAU 1:** Caractéristiques générales des enfants inclus dans l’étude.

Caractéristiques	Lomé	Sokodé	Ensemble
nombre d’enfants inclus	115	98	213
âge moyen ± écart type en mois	35.5 ± 15.2	35.2 ± 14.5	35.3 ± 14.8
sexe (garçon/fille)	67/48	57/41	124/89
poids moyen ± écart type en kg	13.0 ± 3.1	12.7 ± 3.0	12.8 ± 3.0
température moyenne ± écart type en °C	39.1 ± 0.92	38.5 ± 0.92	38.7 ± 0.92

### Étude comparative des deux méthodes

#### Comparaison des deux méthodes selon les valeurs du taux d’hémoglobine

**FIGURE 1:**
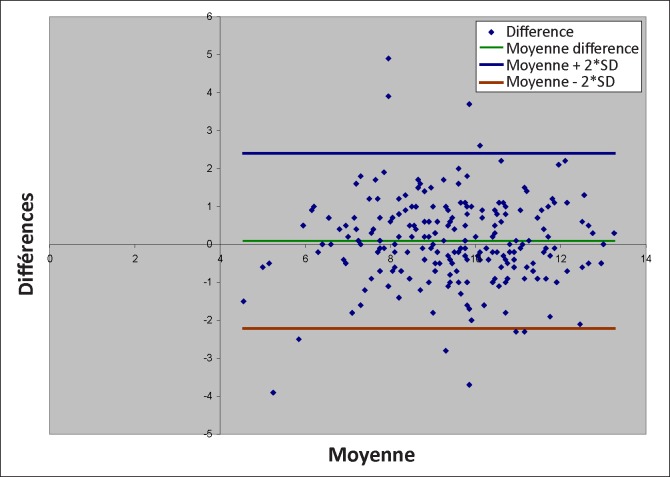
Dispersion des différences entre les valeurs du taux d’hémoglobine par la méthode Hemocue Hb201+® et la méthode analyseurs selon la méthode de Bland et Altman.

Le taux d’hémoglobine moyen était respectivement de 95.0 g/l (±18,1) et de 94.1 g/l (±18.1) pour les deux analyseurs d’hématologie et pour Hemocue Hb201+^®^ (figure 1).

La moyenne des différences entre les valeurs des deux méthodes était de 0.9 ± 11.5 g/l. Les limites considérant 95% des valeurs (± 2 SD) étaient de -22.1 g/l pour la limite inférieure et +23.9 g/l pour la limite supérieure.

Le coefficient de corrélation de Pearson était de 0.80. Le tableau 2 montre la répartition du nombre d’échantillons selon les différences de taux d’hémoglobine entre les deux méthodes.

**TABLEAU 2:** Nombre d’échantillons selon les différences du taux d’hémoglobine entre les deux méthodes.

Méthodes	[-1; +1] *N* (%)	[-2; +2] *N* (%)	[-3; +3] *N* (%)
Nombre d’échantillons	155 (72.77)	193 (90.61)	213 (100.00)

Soixante-douze virgule soixante-dix-sept pour cent des valeurs d’Hemocue Hb 201^®^+ étaient à ±10 g/l de celles de la méthode de référence et 90.6% à ± 20 g/l des mesures de référence (tableau 2).

#### Comparaison des deux méthodes pour le diagnostic de l’anémie

La prévalence de l’anémie était de 81.2% (tableau 3). Autant à Lomé qu’à Sokodé, l’Hemocue Hb 201+^®^ présentait une bonne sensibilité et une spécificité moyenne; 90.2% des patients étaient effectivement anémiés lorsque Hemocue Hb 201+^®^ donnait un taux d’hémoglobine inférieur à 110 g/l (tableau 4).

**TABLEAU 3:** Prévalence de l’anémie à partir des deux méthodes de dosage.

Méthodes	Automate *N* (%)	Hemocue Hb 201+® *N* (%)
**Enfants anémiés**	173 (81.22)	169 (79.34)
**Enfants non anémiés**	40 (18.78)	44 (20.66)

**Total**	**213 (100)**	**213 (100)**

χ^2^ = 0,13 et P = 0,71 (NS)

**TABLEAU 4:** Évaluation de la performance intrinsèque et extrinsèque de l’Hemocue Hb 201+®.

Caractéristiques	Sensibilité		Spécificité		VPP		VPN		Exactitude	
	%	IC	%	IC	%	IC	%	IC	%	IC
**Evaluation à Lomé**	96.8	(90.2-99.2)	59.1	(36.7-78.5)	90.9	(83.0-95.5)	81.3	(53.7-95,8)	89.6	(70.1-91.2)
**Evaluation à Sokodé**	93.0	(83.7-97.4)	70.4	(49.7-85.5)	89.2	(79.3-94.9)	79.2	(57.3-92,1)	86.7	(69.3-89.4)
**Evaluation globale**	95.1	(90.3-97.7)	65.3	(50.3-77.9)	90.2	(84.5-94.0)	80.0	(63.9-90,4)	88.3	(71.0-92.6)

## Discussion

### Contraintes de l’étude

Notre étude étant couplée à une étude déjà réalisée à d’autres fins, certaines informations qui permettaient de faire une évaluation plus complète de la performance d’Hemocue Hb 201+^®^ ont fait défaut. La répétabilité d’Hemocue Hb201+^®^ et des deux analyseurs pour le dosage de l’hémoglobine n’a pas été préalablement évaluée; les limites de variations acceptables ont donc été fixées de façon arbitraire à ±10 g/l. On aurait également pu disposer d’un groupe témoin d’enfants apparemment sains et non anémiés pour faciliter la comparaison.

### Population étudiée

La population d’étude a regroupé 213 enfants des deux sexes âgés de 6 à 59 mois avec une légère prédominance masculine, le poids moyen était de 12.8 kg et la température moyenne de 38.7 °C. Le sex-ratio retrouvé dans notre étude était peut être dû au hasard car avant l’âge de 5 ans tous les enfants, indépendamment du sexe sont prédisposés au paludisme étant donné qu’ils ne sont pas encore pré-immunisés.

Bien que la population d’étude soit inégalement répartie sur les deux sites, le nombre d’enfants participant à l’étude étant plus élevé à Lomé qu’à Sokodé, les caractéristiques sociodémographiques des enfants enrôlés sur les deux sites étaient similaires.

### Analyse comparative des valeurs du taux d’hémoglobine

Il n’existait pas de différence significative entre le taux moyen d’hémoglobine obtenu avec les analyseurs (95 g/l ±18.1) et celui obtenu par Hemocue Hb201+^®^ (94.1 g/l ±18.1). Le coefficient de corrélation de Pearson entre les deux méthodes était de 0.80, démontrant ainsi la proximité des valeurs obtenues avec les deux méthodes. Les limites de dispersion des différences selon la méthode statistique de Bland et Altman, limites entre lesquelles 95% des valeurs des différences se trouvaient, étaient de -22.1 g/l et +23.9 g/l. L’exactitude du test Hemocue Hb201+^®^ était de 88.3%. Ces résultats sont similaires à ceux trouvés par Tayou *et al*. au Cameroun en 2004 pour la détermination de l’hémoglobine chez les donneurs de sang.^8^ Ceux-ci ont trouvé des limites de dispersion de -20.6 g/l à + 13.8 g/l, une exactitude de 93.6% et un coefficient de corrélation de 0.91. Nos résultats diffèrent en revanche de ceux trouvés par Paddle *et al*. en Angleterre en 2002^4^, qui ont trouvé des limites de dispersion plus étroites que les nôtres (-11.6 g/l et + 1.6 g/l).

La prévalence de l’anémie était de 79.3% pour la méthode de référence et de 77.9% pour Hemocue Hb201+^®^. Les prévalences de l’anémie par les deux méthodes ne présentent aucune différence significative.

Soixante-douze virgule soixante-dix-sept pour cent des valeurs d’Hemocue Hb 201+^®^ étaient à ±10 g/l de celles de la méthode de référence. Ces résultats diffèrent de ceux trouvés par Paddle *et al*.^4^: 95.3% de leurs résultats se trouvaient à ±10 g/l de celles de la méthode de référence. Il faut noter que ces derniers ont dû évaluer l’échelle de couleurs développée par l’OMS pour le dosage de l’hémoglobine et comparer les résultats du taux d’hémoglobine obtenus avec Hemocue à ceux obtenus par la méthode des couleurs et ceux déterminés par une méthode de référence, l’analyseur Technicon H3.

### Évaluation du test Hemocue 201+^®^ dans le diagnostic de l’anémie

Hemocue Hb 201+^®^ a montré une sensibilité de 96.8% et de 93%, et une spécificité de 59.1% et de 70.4% respectivement sur les sites de Lomé et de Sokodé. Sur l’ensemble des deux sites d’étude, Hemocue Hb 201+^®^ présentait une sensibilité de 95.1% et une spécificité de 65.3%. Ces résultats sont similaires à ceux obtenus par Neville à Dundee (en Écosse) en 1987^9^, qui a obtenu une sensibilité et une spécificité respective de 88.5% et 77.6% dans une étude comparative de la méthode Hemocue avec l’analyseur automatique ELT 80WS (Ortho Diagnostic Systems Ltd) chez 235 patients recrutés dans un centre de santé.

D’autres études ont rapporté une très bonne spécificité de la méthode Hemocue Hb201+^®^: 97.1% pour Tayou *et al*. en 2004 au Cameroun^8^ et 94.2% pour Sari *et al*. en 1998 en Indonésie.^10^ Au Cameroun, l’automate Celly 70 a été utilisé comme méthode de référence et en Indonésie, Hemocue Hb201+^®^ a été comparé à la méthode directe de la cyanméthémoglobine. Cette différence avec nos résultats peut être due au grand nombre de faux positifs (17) trouvés par rapport à la référence choisie. Ceci peut également être dû au fait que l’utilisation de la 3^e^ ou 4^e^ goutte lors de la manipulation n’a pu être rigoureusement respectée. De plus, les conditions d’utilisation d’Hemocue Hb 201+^®^ assez restrictives peuvent également être incriminées: en effet, les microcuvettes d’Hemocue Hb 201+^®^, qui contiennent des réactifs, ne peuvent pas résister à une température supérieure à 30 °C et doivent être utilisées dans un court délai après ouverture. La nouvelle génération de cet équipement, Hemocue Hb 301+^®11^ serait davantage adaptée aux réalités des laboratoires de niveau périphérique, puisque ses microcuvettes, qui ne contiennent pas de réactif, résistent à une température pouvant aller jusqu’à 40 °C, et sont relativement moins coûteuses et donc plus abordables. D’autres études doivent être réalisées en vue d’évaluer Hemocue Hb 301+^®^ pour le dosage de l’hémoglobine dans nos structures sanitaires.

## Conclusion

Les résultats de l’étude ont montré que le test Hemocue Hb201+^®^ présentait une bonne sensibilité, une spécificité moyenne et une exactitude moyenne tant dans le diagnostic de l’anémie que dans le dosage de l’hémoglobine. Son utilisation peut être recommandée dans les structures périphériques afin de faciliter le diagnostic biologique de l’anémie et sa prise en charge dans les populations vivant dans les zones difficiles d’accès.
